# High prevalence of *Acanthamoeba* spp. in agricultural soil of barberry farms: a public health concern in eastern Iran

**DOI:** 10.1016/j.parepi.2026.e00541

**Published:** 2026-09-09

**Authors:** Mahdieh Rezaei-Shirkhond, Mahmood Reza Behravan, Ehsan Javanmard, Maryam Niyyati, Amir Tavakoli Kareshk, Hossein Pazoki, Abdol Sattar Pagheh

**Affiliations:** a Student Research Committee, Birjand University of Medical Sciences, Birjand, Iran; b Infectious Diseases Research Center, Birjand University of Medical Sciences, Birjand, Iran; c Department of Parasitology and Mycology, School of Medicine, Shiraz University of Medical Sciences, Iran; d Department of Parasitology and Mycology, School of Medicine, Shahid Beheshti University of Medical Sciences, Tehran, Iran; e Department of Medical Microbiology, Faculty of Medicine, Infectious Diseases Research Center, Gonabad University of Medical Science, Gonabad, Iran

**Keywords:** *Acanthamoeba* spp., Barberry farms, Molecular analysis, T4 genotypes, Gene flow

## Abstract

*Acanthamoeba* spp. are ubiquitous environmental amoebae and opportunistic pathogens responsible for severe human infections such as keratitis and encephalitis. Their resilient cysts accumulate in soil, creating a reservoir of public health concern, especially in agricultural regions with high human activity. This study presents the first global assessment of *Acanthamoeba* spp. prevalence, genotypes, and phylogenetics in barberry agricultural soils. We collected soil samples from barberry fields in Birjand, Iran. *Acanthamoeba* spp. were isolated by filtration and cultured on non-nutrient agar with *E. coli*, then identified microscopically. DNA was extracted from positive cultures, and a diagnostic fragment of the 18S rRNA gene was amplified using JDP primers for sequencing and genotyping. Phylogenetic and population genetics analyses (Tajima's D, haplotype network) were performed using MEGA11 and DnaSP software. *Acanthamoeba* spp. were identified in 65 of 164 soil samples (39.6%). PCR confirmed all isolates, with sequencing of 10 isolates revealing exclusive T4 genotype identification. Population genetics (π = 0.02079, Tajima's D = 2.46922) indicated balancing selection within this population. Haplotype network analysis demonstrated genetic proximity between these soil isolates and strains from clinical (keratitis) and aquatic sources. This confirms a shared, circulating T4 population in the region. These findings highlight the urgent need for targeted public health interventions in endemic agricultural regions.

## 1 Introduction

Free-living amoebae (FLA) are ubiquitous protozoa widely distributed in soil, freshwater, dust, and various natural and man-made aquatic environments (Rodríguez-Zaragoza, 1994). These organisms exhibit a biphasic life cycle consisting of an active trophozoite stage and a highly resistant cyst stage (Siddiqui and Khan, 2012; Wang et al., 2023). The cyst form enables long-term survival under adverse environmental conditions and facilitates transmission (Javanmard et al., 2017). Among FLA, the genus *Acanthamoeba* is of particular medical and public health importance. *Acanthamoeba* spp. are opportunistic pathogens capable of causing severe human infections, including sight-threatening *Acanthamoeba* keratitis (AK) primarily in contact lens wearers and rare but fatal granulomatous amoebic encephalitis (GAE), as well as disseminated cutaneous infections, especially in immunocompromised individuals (Lorenzo-Morales et al., 2015; Anwar et al., 2018; Garg and Daigavane, 2024). The widespread presence of *Acanthamoeba* in soil environments underscores its importance in natural ecosystems; however, it also raises concerns about its potential impact on human health (Geisen et al., 2014). The detection and identification of *Acanthamoeba* spp. in soil are of paramount importance, not only for understanding ecological dynamics but also for public health considerations within the framework of the One Health approach (Siddiqui et al., 2022). One Health recognizes the interconnectedness of human, animal, and environmental health, emphasizing that environmental reservoirs, such as soil, can serve as collective sources for pathogenic agents, including certain genotypes of *Acanthamoeba*. spp. (Calistri et al., 2013; Who, 2017).

Traditional methods include microscopic examination of trophozoite and cyst morphology, which provides initial identification but often lacks specificity (Zhang et al., 2023). Molecular techniques, particularly polymerase chain reaction (PCR) targeting the 18S rRNA gene, and the amplification of a discriminative fragment (DF3 region) are common practices to determine the genus, species, and genotypes (Risler et al., 2013), so far allowing for highly sensitive and specific identification of 23 *Acanthamoeba* genotypes (Otero-Ruiz et al., 2022). The presence of *Acanthamoeba* spp. in soil environments acts as a potential vehicle for transmitting pathogenic microorganisms, as these protozoa can harbor bacteria (*Legionella* spp.and *Mycobacterium* spp.), viruses within their trophozoites and cysts a phenomenon that has significant implications for disease transmission (Adékambi et al., 2006; Bouyer et al., 2007).

Consequently, understanding the prevalence, diversity, and genetic makeup of *Acanthamoeba* spp. in barberry fields is crucial for implementing effective environmental monitoring and risk assessment strategies. To the best of our knowledge, this study, being the first of its kind globally in barberry fields, highlights the importance of addressing environmental health in agricultural settings. By considering the principles of One Health, which integrates human and environmental health, this research emphasizes the interconnectedness of ecosystem health and its potential impact on human health, particularly in areas with high human activity (Humboldt-Dachroeden and Mantovani, 2021; Ma et al., 2023). The purpose of this study is to assess the prevalence of *Acanthamoeba* spp. in the soil of barberry farms associated with high human activity in South Khorasan Province, eastern Iran.

## 2 Materials and methods

### 2.1 Study area

**Fig. 1:**
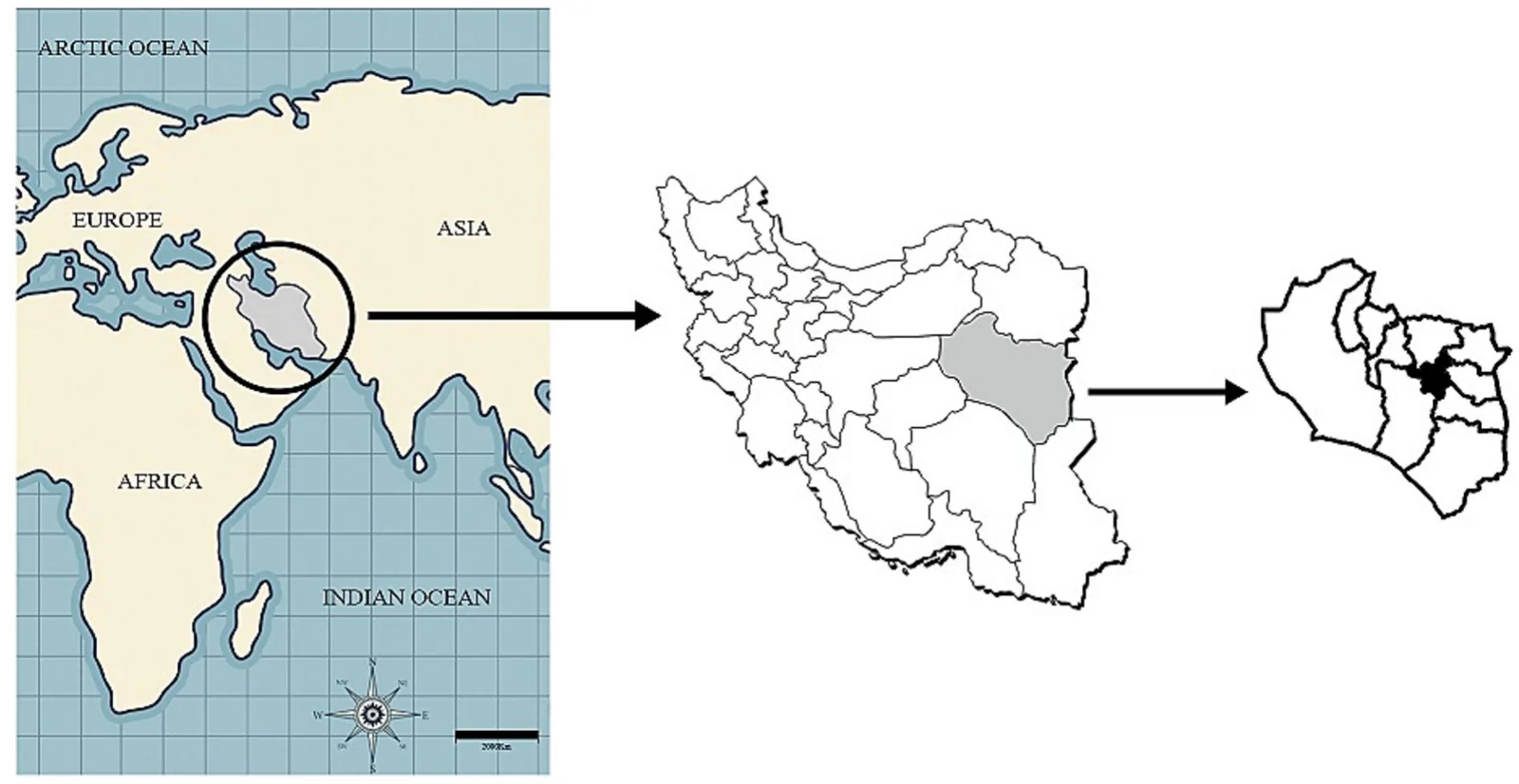
illustrates the map of South Khorasan Province, highlighting the sampling sites selected for this research.

Soil samples (*n* = 164) were collected from four geographically distinct barberry cultivation sites within Birjand city, South Khorasan province, Iran. The city is renowned for its exports of barberry, Saffron, and jujube. According to the 2016 census, the city's population stands at 203,636 people living in 57,745 households (Fig. 1). The study protocol was reviewed and approved by the Ethics Committee of Birjand University of Medical Sciences (Approval Code: IR.IUMS.REC.1402.435).

### 2.2 Sample processing

This cross-sectional study was conducted in 2024 by collecting soil samples from barberry fields in Birjand city. Samples were obtained by directly extracting 50 g of soil at a depth of 2–3 cm. All samples were subsequently transferred to the Microbiology Laboratory at the Faculty of Medicine, Birjand University of Medical Sciences, for the specified analyses. Sample filtration was performed using a vacuum pump, 0.45-μm nitrocellulose filters, and sterile distilled water to dissolve 50 g of soil. Filter paper containing sediments was placed sediment-side down on 1.2% Non-nutrient agar (NNA, Difco, Sparks, MD, USA), along with a lawn of inactivated *Escherichia coli* strain K12 (Fatemi et al., 2023). All plates were incubated at room temperature (26 °C ± 3 °C) and monitored every 48 h for *Acanthamoeba* spp. growth over one month. Positive plates were then sub-cultured to achieve a pure culture of the targeted amoebae. Plates were then sealed with parafilm to prevent the samples from drying out. For subculturing, parts of the NNA containing trophozoites/cysts free of fungal and bacterial contamination were transferred to new plates (Chomicz et al., 2023). Incubation temperature and duration were same as primary cultures (Fatemi et al., 2023). The presence of *Acanthamoeba* spp. was initially confirmed by observing characteristic morphological features according to Page (1988), followed by definitive identification using molecular methods.

### 2.3 DNA extraction and PCR amplification

*Acanthamoeba* spp. were harvested following previously established protocols (Mahdavi et al., 2024). Briefly, plates were washed with 5 mL of sterile phosphate-buffered saline (PBS; pH = 7.2), and the amoebae were concentrated by centrifugation at 2000 rpm for 5 min. After that, the supernatant was discarded, and DNA was extracted from the remaining pellet using a total nucleic acid extraction kit (Pars Toos Company Kit, Iran). The concentration and purity of the extracted DNA were assessed by measuring the optical density (OD) at 260 nm and 280 nm using a Nano-Drop 2000™ spectrophotometer (Thermo Fisher Scientific, USA). PCR amplification was conducted using specific primers including JDP1 (5′-GGCCCAGATCGTTTACCG TGAA-3′) and JDP2 (5′-TCTCACAAGCTGCTAGGGGATA-3′) for *Acanthamoeba* spp. (Schroeder et al., 2001). PCR reactions were performed in a total volume of 15 μL containing 7.5 μL of 2× red master mix (Ampliqon, Denmark), 10 ρmol of each primer, DNA (at least 10 ng), and distilled water. The thermal cycling profile was 94 °C for 3 min, followed by 35 repeated cycles, including 94 °C for 35 s, 56 °C for 45 s, and 72 °C for 1 min. The final extension cycle was performed at 72 °C for 5 min (Mahdavi et al., 2024). A sequenced isolate for was employed as positive control (Acc. Nos. PP659719.1 for *Acanthamoeba*. PCR amplification of the target was verified through visualization of the products on a 1.5% agarose gel stained with Safe Stain and observed under UV light. The genetic identity of 10 amplified PCR products was determined by Sanger sequencing using an ABI 3130 system (Applied Biosystems, California, USA).

### 2.4 Genotyping and phylogenetic analysis

Sequence editing and visualization were performed using Chromas version 2.6.6, followed by alignment using ClustalW, which is integrated into BioEdit version 7.0.9. Ambiguous sites were verified against existing sequences in the GenBank database. Sequence comparison with the GenBank database was carried out using the Basic Local Alignment Search Tool (BLAST) to ascertain their genotypes. All sequences were submitted to GenBank under accession numbers PV596404- PV596413. Aligned sequences were screened based on a consensus sequence to identify possible gaps and subsequently trimmed. (This analysis involved 29 nucleotide sequences. There were a total of 238 positions in the final dataset. Evolutionary analyses were conducted in MEGA11) (Tamura et al., 2021).

The Bayesian Information Criterion was applied to select the optimal phylogenetic tree based on the model nucleotide substitution. The Maximum-Likelihood algorithm and the Tamura 3-Parameter option were utilized within the Molecular Evolutionary Genetics Analysis (MEGA11) software for phylogenetic analysis. Bootstrap with 1000 replications was executed to assess statistical support for distance (Tamura et al., 2021).

### 2.5 DnaSP and network analyses

In order to investigate the genetic relationship between the sequences obtained in this study and those previously isolated from various sources such as river water, soil, AK patients, and hospital wastewater, DnaSP v5 (Librado and Rozas, 2009) was utilized. Haplotype diversity (Hd), segregation sites (S), nucleotide diversity (π), gene flow, and Tajima's D test were computed for *Acanthamoeba*-positive samples to further analyze their genetic characteristics.

Network analysis utilizing PopART was carried out to explore potential infection sources and evaluate their distribution and adaptability across diverse ecological niches (Leigh et al., 2015). For this purpose, the Transitive/Tree-Based Consistency Score (TCS) method was employed to construct a network based on parsimony analysis of the aligned sequences (Clement et al., 2000). This method enables visualization of the genetic relationships both within and between species, providing insights into their evolutionary pathways, species diversity, and potential transmission dynamics. In the context of this study, the genetic diversity and relationships among *Acanthamoeba* spp. genotypes derived from various sources were investigated, contributing to a better understanding of the complexity and interconnectedness of these organisms within different environments.

## 3 Results

### 3.1 Microscopy and culture analysis

**Fig. 2:**
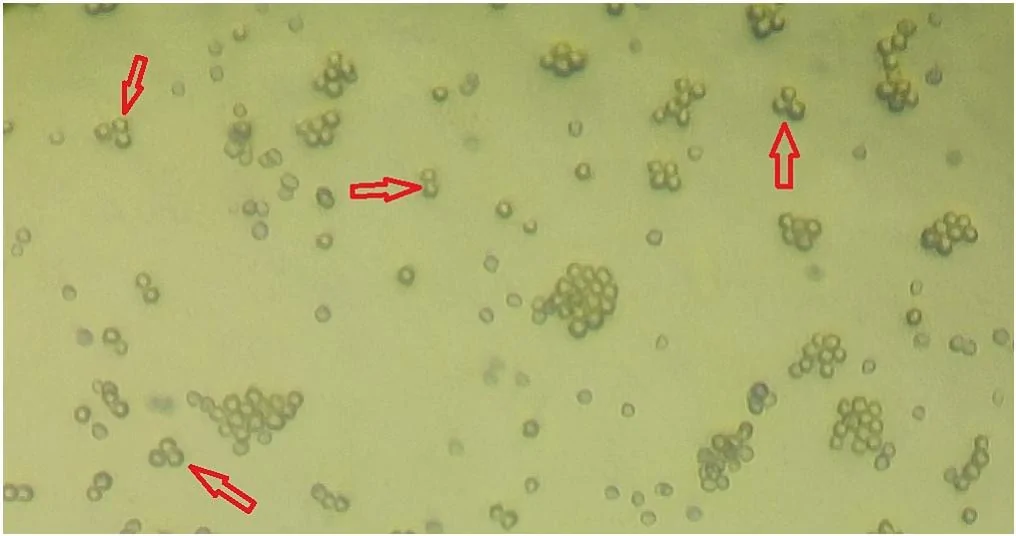
Microscopic photographs showing cysts of *Acanthamoeba* spp. with predominantly polygonal shapes on the NNA plate. Red-filled arrows point to the polygon-shaped cysts. The images are magnified at 400 × . (For interpretation of the references to colour in this figure legend, the reader is referred to the web version of this article.)

Culture-based characterization showed that 65/164 (39.6%) of plates were positive for FLA (Table 1). Microscopic examination revealed that these positive samples contained *Acanthamoeba* spp. cysts with predominantly polygonal shapes (Table 1, Fig. 2). Morphometric measurements and morphological group assignments were not performed, as molecular methods (PCR and sequencing) were considered the definitive and primary identification approach in this study.

**Table 1:** Characteristics of positive samples based on culture and microscopy methods.

Geographical areas		Total number of samples	Number of positive samples	Culture-positive	Total positive samples
North	Abbasabad-e dasht	10	3	4	24
	Hajjiabad, Alqurat	10	8	8	
	Arviz	12	7	8	
	Hambal	10	6	6	
South	Khunik	12	4	5	12
	Khorashad	10	4	5	
	Barzej	6	2	2	
	Surg	12	2	2	
East	Rahnishk	10	5	6	15
	Sar Chah-e Tazian	10	2	3	
	Asiaban	10	3	3	
	Gomach	8	2	3	
West	Shir Khond	12	3	5	14
	Nowkand	10	5	5	
	Khunik-e mazar	12	6	8	
	Givak-e Sofla	10	3	3

### 3.2 Molecular analysis

**Fig. 3:**
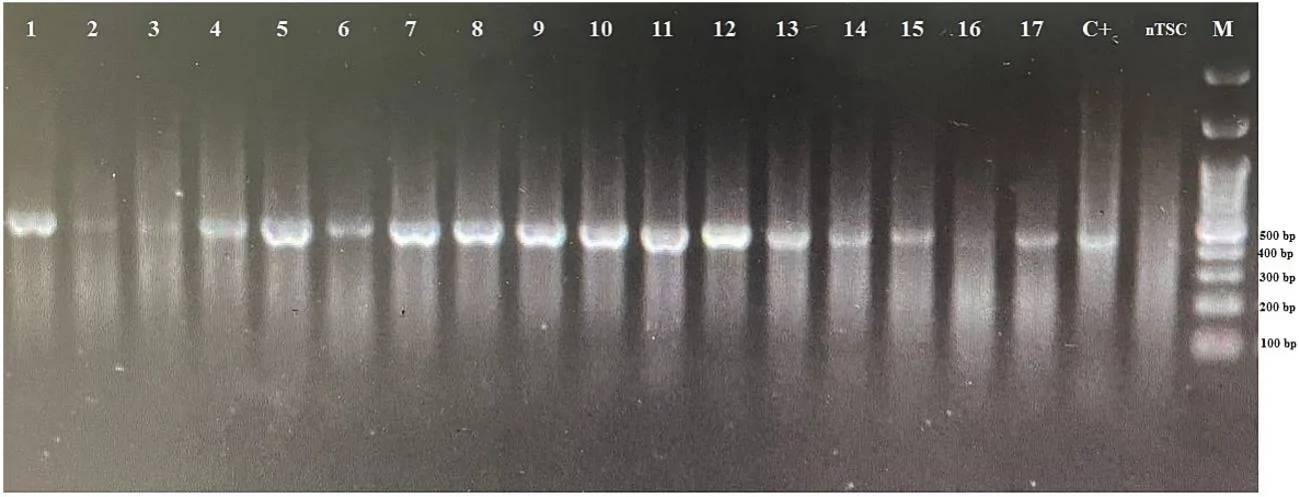
Agarose gel electrophoresis of PCR-amplified products of the 18S rRNA gene (∼450 bp) from *Acanthamoeba* spp. isolated from soil samples of barberry farms in Birjand city. M: 100 bp DNA ladder; C+: Positive control; nTSC: non-target specificity control; lanes 1–17: representative positive samples.

All microscopic positive samples were subsequently analysed using a PCR assay. Of the 65 samples identified microscopically as *Acanthamoeba* spp., a 450-bp fragment specific to *Acanthamoeba* was amplified (Fig. 3). Ten isolates were randomly selected for sequencing, and all were identified as genotype T4 with 99% homology (accession numbers PV596404–PV596413) (Table 2). The quality and quantity of the extracted DNA were assessed by measuring the optical density (OD) at 260 nm, 280 nm, and 230 nm using a Nano Drop spectrophotometer. The DNA concentration of the 10 sequenced isolates ranged from 52.9 to 85.4 ng/μL, with OD260/OD280 ratios between 1.82 and 1.98 and OD260/OD230 ratios between 1.91 and 2.15, indicating high purity and the absence of significant protein or salt contamination (Supplementary Table S1a).

**Table 2:** Molecular detection and genotypes of culture-positive samples.

No	Geographical areas		Culture-positive	Molecular results (Genotype)	Accession number
1	North	Hambal	*Acanthamoeba spp*	T4	PV596404
2	East	Shirkhond	*Acanthamoeba spp*	T4	PV596405
3	North	Arviz	*Acanthamoeba spp*	T4	PV596406
4	North	Arviz	*Acanthamoeba spp*	T4	PV596407
5	South	Khunik	*Acanthamoeba spp*	T4	PV596408
6	South	Khunik	*Acanthamoeba spp*	T4	PV596409
7	North	Hajjiabad	*Acanthamoeba spp*	T4	PV596410
8	North	Arviz	*Acanthamoeba spp*	T4	PV596411
9	North	Arviz	*Acanthamoeba spp*	T4	PV596412
10	East	Sar Chah-e Tazian	*Acanthamoeba spp*	T4	PV596413

### 3.3 DnaSP analysis

**Fig. 4:**
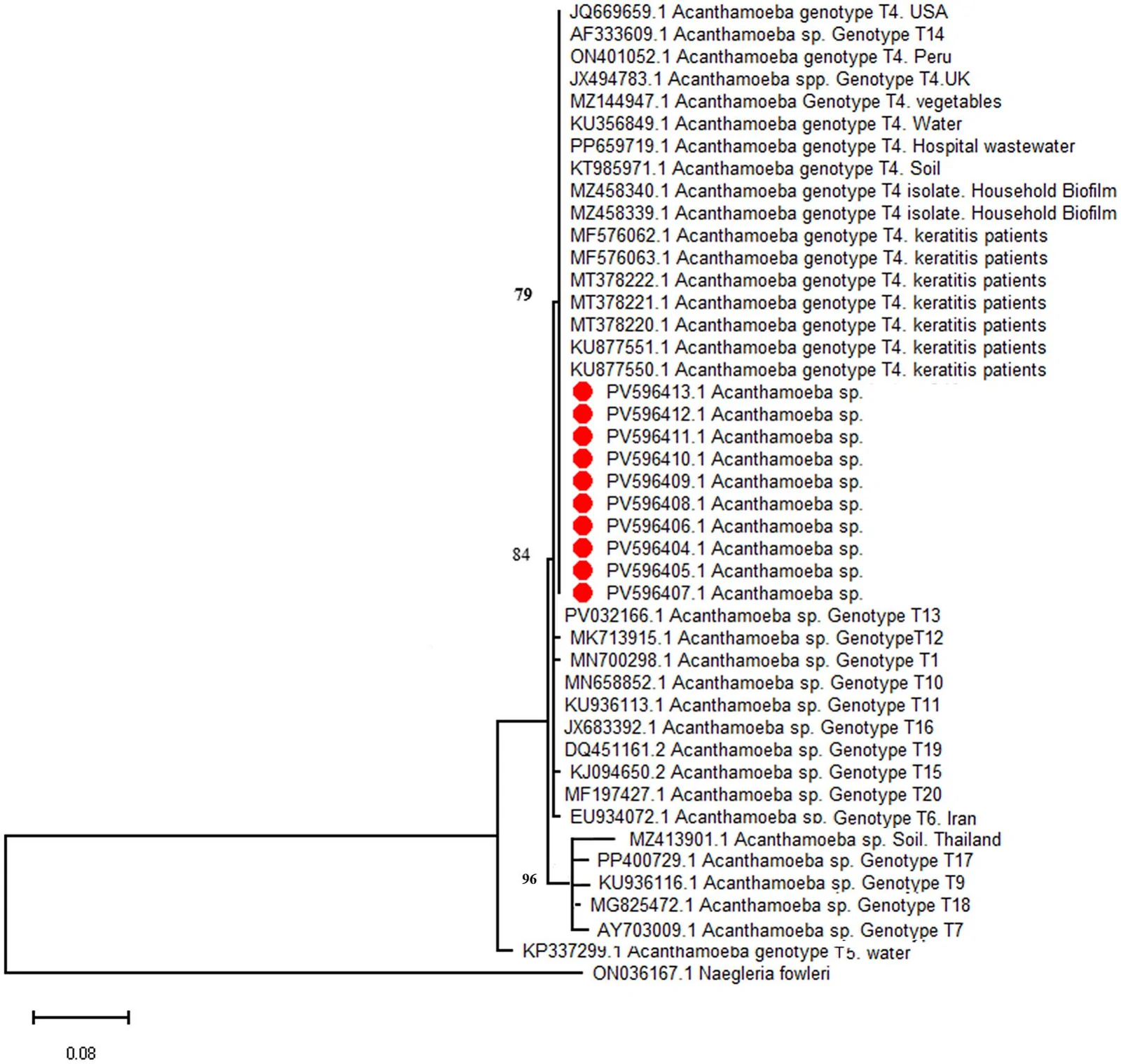
Phylogenetic tree of *Acanthamoeba* genotypes T4 was constructed through maximum likelihood analysis using Tamura's 3-parameter model for nucleotide substitutions. Sequence alignment was confirmed with 1000 bootstrap replicates. Study sequences are marked in red. (For interpretation of the references to colour in this figure legend, the reader is referred to the web version of this article.)

Five haplotypes were detected within genotype T4, which was more diverse compared to other genotypes. DnaSP analysis revealed a π index of 0.02079 among genotype T4. Additionally, there were 13 segregating sites and 13 mutations. Haplotype diversity (Hd) was calculated to be 0.00940. The tests indicated a positive Tajima's D value of 2.46922 for genotype T4. Our results showed gene flow between soil-derived T4 genotype isolates (PV596404-PV596413) and T4 sequences recovered from Birjand water sources (PV607986-PV607996). the comparison of the index in other populations suggests close genetic relationship between the T4 genotypes (Fig. 4).

### 3.4 Haplotype networking and phylogenetic analysis

**Fig. 5:**
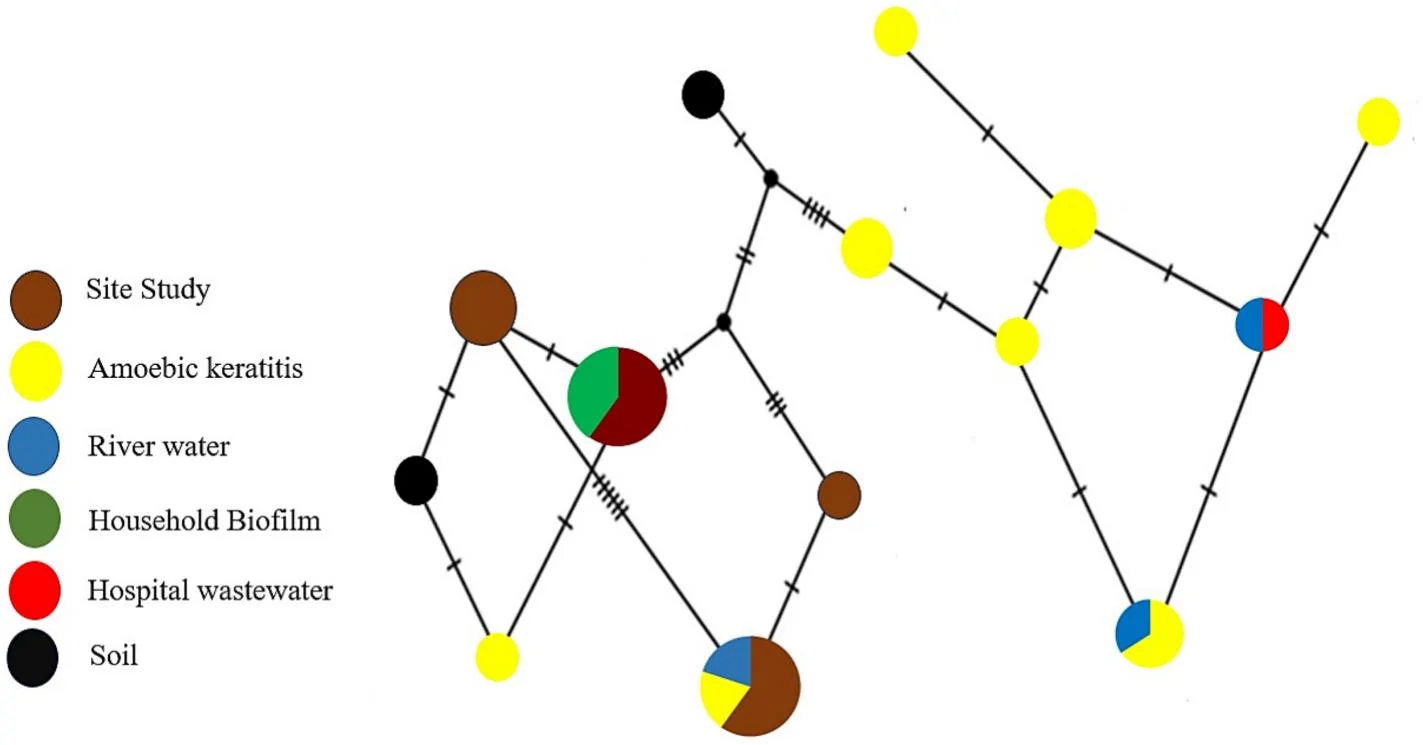
The TCS sequence-type network was created for the same fragment of the 18S rRNA gene. The size of each circle reflects the relative abundance of sequences within each haplotype. Mutations are represented by hashes along the branches of the network.

Networking confirmed the findings of the phylogenetic analysis and showed the presence of three distinct haplotype types among soil isolates of *Acanthamoeba* spp. with one or more hash marks along the lines, which indicate the mutation steps (genetic distances). The haplotypes of the T4 genotype of our study were placed next to the haplotypes from the T4 genotype of the previous studies, which were collected from AK patients (Nayeri Chegeni et al., 2019; Esboei et al., 2020), household biofilm (Norouzi et al., 2021), and River water (Javanmard et al., 2025) isolates, supporting close genetic relationship, which was suggested by the gene flow analysis (Fig. 5).

## 4 Discussion

As emerging opportunistic pathogens, *Acanthamoeba* spp., represent a growing public health challenge, especially when prevalent in high-contact environments like agricultural soil (Reyes-Batlle et al., 2021). In this study, we detected *Acanthamoeba* spp. in 39.6% of soil samples from barberry orchards in South Khorasan Province, Iran.

Our finding is consistent with prevalence rates documented in Tehran (∼30%) (Pazoki et al., 2020) and Khuzestan (26%) (Rahdar et al., 2012), corroborating the extensive environmental presence of this pathogen in Iran. It is, however, lower than the 41.6% reported in East Azerbaijan (Karamati et al., 2016) and considerably lower than the 100% prevalence documented in the humid, subtropical climate of Gilan Province (Mahmoudi et al., 2022). These pronounced regional disparities are likely attributable to key ecological determinants such as soil moisture, organic content, microbial competition, and broader climatic factors, compounded by variations in sampling and detection methodologies. Notably, a study conducted in Birjand a decade earlier reported *Acanthamoeba* contamination in 39% of recreational water sources using culture and morphological identification (Behravan et al., 2015). The striking similarity in prevalence between that study and the current molecular-based investigation, despite the 10-year gap, suggests persistent environmental contamination in this region. Internationally, soil contamination rates have ranged widely: 40–62.5% in the Canary Islands (Reyes-Batlle et al., 2014). 63.9% in Jamaica (Todd et al., 2015). and up to 58.8% in southeastern Iran and neighboring Pakistan (Tanveer et al., 2015).

Consistent with different studies, genotype T4 the predominant lineage in environmental samples, especially from soils with high anthropogenic activity (Badirzadeh et al., 2011; Kialashaki et al., 2018; Aykur and Dagci, 2023), was the sole genotype identified in our ten sequenced isolates. Critically, T4 is the genotype most frequently implicated in human pathologies, including *Acanthamoeba* keratitis and granulomatous amoebic encephalitis (Niyyati et al., 2009; Tanveer et al., 2013; Megha et al., 2018; Esboei et al., 2020). This genetic characterization, therefore, confirms that the *Acanthamoeba* reservoir in these barberry farms is of direct public health relevance. Furthermore, a significantly positive Tajima's D value (2.46922) further supports the hypothesis of balancing selection or a recent population bottleneck, contrasting with negative values reported in more humid regions (Tajima, 1989; Spotin et al., 2017). These findings suggest that T4 populations in arid agricultural soils may follow distinct evolutionary trajectories compared to those in wetter environments.

Phylogenetic and haplotype network analyses provided compelling evidence of genetic connectivity between soil isolates from barberry fields and previously reported T4 sequences from clinical AK cases (Nayeri Chegeni et al., 2019) and household biofilms (Norouzi et al., 2021). The haplotype network analysis in this study demonstrated that *Acanthamoeba* sequences found in soil share haplotypes with sequences from river water (PQ056828), and keratitis patients (KU877550). This suggests a potential genetic relationship between *Acanthamoeba* T4 sequences across different sources, potentially indicating shared transmission pathways or common environmental factors that influence their genetic composition. The low genetic differentiation between the soil sequences identified in this study and the water source sequences previously reported in Birjand city provides further evidence of the active transmission cycle of *Acanthamoeba* spp. in different sources. This suggests that the interaction between populations of *Acanthamoeba* spp. in soil and water may contribute to their evolutionary dynamics and adaptation to different environments (Javanmard et al., 2025). This indicates that the movement of genes between *Acanthamoeba* spp. populations in soil and water may contribute to their evolutionary dynamics and adaptation to different environments.

Barberry cultivation in South Khorasan is notably drought-tolerant, with orchards typically established near scarce water sources. These sites become focal points for frequent human activity by farmers and workers (Moradinezhad et al., 2025). Our findings, which confirm the presence of pathogenic T4 genotypes in this environment, underscore an urgent need for parallel clinical and epidemiological studies in the region. Such research is critical to delineate the precise transmission routes from this agricultural reservoir to human hosts. This interconnectedness between a resilient agricultural practice, a contaminated environment, and a high-risk human population exemplifies the necessity of a One Health framework. Only through integrated research spanning environmental, agricultural, veterinary, and human medical disciplines can we fully decipher the complex lifecycle and public health threat of free-living amoebae like *Acanthamoeba* spp.

## 5 Conclusion

This study confirms that the agricultural soils of South Khorasan barberry orchards constitute a significant environmental reservoir for *Acanthamoeba* spp. Critically, phylogenetic and haplotype network analyses demonstrated a close genetic relationship between our environmental T4 strains and those isolated from *Acanthamoeba* keratitis patients and hospital wastewater, suggesting a shared, circulating population. These results establish a clear epidemiological link between this agricultural niche and potential human infection pathways. Consequently, the routine agricultural practices in these settings, characterized by intensive human-soil contact, transform barberry farming into an occupation with a tangible risk of exposure to pathogenic *Acanthamoeba* spp. Our findings underscore the necessity of integrating environmental surveillance with public health strategies in endemic regions, highlighting the value of a One Health approach to mitigate the risk of opportunistic infections from free-living amoebae.

## CRediT authorship contribution statement

**Mahdieh Rezaei-Shirkhond:** Writing – review & editing, Methodology. **Mahmood Reza Behravan:** Writing – review & editing, Writing – original draft, Methodology. **Ehsan Javanmard:** Writing – review & editing, Writing – original draft. **Maryam Niyyati:** Writing – review & editing. **Amir Tavakoli Kareshk:** Writing – review & editing. **Hossein Pazoki:** Writing – review & editing, Writing – original draft, Methodology. **Abdol Sattar Pagheh:** Writing – review & editing, Writing – original draft, Supervision, Methodology, Conceptualization.

## Ethics approval

The study protocol was reviewed and approved by the Ethics Committee of Birjand University of Medical Sciences (Approval Code: IR.IUMS.REC.1402.435).

## Funding

This work was supported by 10.13039/501100005117Birjand University of Medical Sciences, Birjand, Iran (Grant Number 457282).

## Declaration of competing interest

The authors declare that they have no known competing financial interests or personal relationships that could have appeared to influence the work reported in this paper.

## Data Availability

The data that support the findings of this study are available from the corresponding author upon reasonable request.
