# Supplementary material for: High prevalence of Acanthamoeba spp. in agricultural soil of barberry farms: a public health concern in eastern Iran
Source: Parasite Epidemiol Control. 2026 Sep 9;35:e00541. doi: 10.1016/j.parepi.2026.e00541 (PMC13590051; doi:10.1016/j.parepi.2026.e00541)
Supplement: Supplementary file 1 [file mmc1.docx]

| **Isolate Accession** | **DNA Concentration (ng/µL)** | **A260/A280 Ratio** | **A260/A230 Ratio** |
| --- | --- | --- | --- |
| PV596404 | 85.4 | 1.89 | 2.11 |
| PV596405 | 79.7 | 1.82 | 1.95 |
| PV596406 | 83.8 | 1.84 | 2.01 |
| PV596407 | 61.1 | 1.88 | 2.12 |
| PV596408 | 52.9 | 1.87 | 1.92 |
| PV596409 | 75.4 | 1.95 | 2.12 |
| PV596410 | 67.1 | 1.98 | 2.12 |
| PV596411 | 82.5 | 1.93 | 2.15 |
| PV596412 | 76.3 | 1.82 | 1.98 |
| PV596413 | 64.7 | 1.91 | 1.91 |

**Supplementary Table S1a.** DNA concentration and purity ratios of the 10 sequenced Acanthamoeba T4 isolates (accession numbers PV596404–PV596413) from barberry farm soil samples, measured by NanoDrop spectrophotometer.

Supplementary Table S1b. DNA concentration and purity ratios of the 65 *Acanthamoeba*-positive isolates from barberry farm soil samples, measured by NanoDrop spectrophotometer.

| **Isolate ID** | **DNA Concentration (ng/µL)** | **A260/A280 Ratio** | **A260/A230 Ratio** |
| --- | --- | --- | --- |
| 1 | 26.0 | 1.90 | 2.12 |
| 2 | 14.6 | 1.89 | 2.04 |
| 3 | 11.8 | 1.84 | 2.01 |
| 4 | 61.1 | 1.88 | 2.12 |
| 5 | 90.5 | 1.85 | 1.90 |
| 6 | 82.5 | 1.93 | 2.15 |
| 7 | 79.5 | 1.81 | 1.86 |
| 8 | 62.9 | 1.92 | 1.82 |
| 9 | 78.8 | 1.66 | 1.59 |
| 10 | 63.5 | 1.94 | 1.79 |
| 11 | 82.5 | 1.88 | 1.82 |
| 12 | 90.6 | 1.96 | 2.10 |
| 13 | 76.3 | 1.82 | 1.98 |
| 14 | 91.1 | 1.88 | 1.86 |
| 15 | 80.2 | 1.90 | 1.98 |
| 16 | 12.9 | 1.87 | 1.92 |
| 17 | 27.5 | 1.94 | 1.93 |
| 18 | 33.7 | 1.82 | 1.86 |
| 19 | 46.4 | 1.85 | 1.87 |
| 20 | 71.3 | 1.87 | 1.77 |
| 21 | 39.9 | 1.87 | 1.74 |
| 22 | 66.4 | 1.86 | 1.76 |
| 23 | 31.8 | 1.78 | 2.05 |
| 24 | 58.3 | 1.82 | 2.14 |
| 25 | 59.4 | 1.79 | 1.89 |
| 26 | 45.8 | 1.79 | 1.76 |
| 27 | 64.7 | 1.91 | 1.91 |
| 28 | 74.2 | 1.77 | 1.65 |
| 29 | 44.6 | 1.92 | 2.14 |
| 30 | 48.3 | 1.85 | 2.14 |
| 31 | 75.4 | 1.95 | 2.12 |
| 32 | 61.3 | 1.96 | 1.86 |
| 33 | 74.7 | 1.86 | 1.74 |
| 34 | 56.2 | 1.84 | 1.99 |
| 35 | 32.5 | 1.98 | 1.92 |
| 36 | 52.0 | 1.79 | 1.98 |
| 37 | 94.9 | 1.95 | 1.97 |
| 38 | 87.6 | 1.77 | 1.85 |
| 39 | 37.6 | 1.76 | 1.73 |
| 40 | 55.1 | 1.76 | 1.94 |
| 41 | 48.5 | 1.79 | 2.00 |
| 42 | 63.4 | 1.92 | 1.84 |
| 43 | 67.1 | 1.98 | 2.12 |
| 44 | 82.1 | 1.81 | 1.86 |
| 45 | 36.7 | 1.87 | 1.87 |
| 46 | 81.0 | 1.88 | 1.81 |
| 47 | 45.9 | 1.97 | 2.15 |
| 48 | 63.6 | 1.79 | 1.98 |
| 49 | 79.7 | 1.82 | 1.95 |
| 50 | 47.4 | 1.93 | 1.84 |
| 51 | 76.5 | 1.68 | 1.74 |
| 52 | 69.5 | 1.75 | 1.61 |
| 53 | 61.3 | 1.93 | 1.86 |
| 54 | 65.4 | 1.86 | 1.79 |
| 55 | 40.1 | 1.87 | 1.70 |
| 56 | 26.4 | 1.80 | 1.96 |
| 57 | 55.4 | 1.86 | 1.72 |
| 58 | 63.9 | 1.83 | 2.10 |
| 59 | 87.3 | 1.83 | 1.76 |
| 60 | 58.4 | 1.95 | 2.02 |
| 61 | 64.6 | 1.90 | 2.02 |
| 62 | 85.4 | 1.89 | 2.11 |
| 63 | 67.0 | 1.72 | 1.70 |
| 64 | 52.5 | 1.98 | 1.73 |
| 65 | 63.3 | 1.94 | 1.80 |
